# *“I Would Rather Take the Vaccine Than Undergo Weekly Testing”*: Correlates of Health Workers’ Support for COVID-19 Vaccine Mandates

**DOI:** 10.3390/ijerph192113937

**Published:** 2022-10-26

**Authors:** Zubairu Iliyasu, Rayyan M. Garba, Mansur A. Aliyu, Auwalu U. Gajida, Taiwo G. Amole, Amina A. Umar, Hadiza M. Abdullahi, Fatimah I. Tsiga-Ahmed, Aminatu A. Kwaku, Meira S. Kowalski, Hamisu M. Salihu, Muktar H. Aliyu

**Affiliations:** 1 Department of Community Medicine, Bayero University, Kano PMB 3011, Nigeria; 2 Medicine, Health, and Society Program, Vanderbilt University, Nashville, TN 37240, USA; 3 Department of Family and Community Medicine, Baylor College of Medicine, Houston, TX 77030, USA; 4 Department of Health Policy, Vanderbilt Institute for Global Health, Vanderbilt University Medical Center, Nashville, TN 37203, USA

**Keywords:** COVID-19 response, vaccine mandates, healthcare workers

## Abstract

This study examined the support for vaccine mandates and uptake among clinical and non-clinical staff at a tertiary hospital in northern Nigeria, focusing on variation of survey responses based on job position, socio-demographic characteristics, and perceived risk of infection with severe acute respiratory syndrome coronavirus-2 (SARS-CoV-2). Using an explanatory, sequential, mixed-methods design and deploying a pragmatic paradigm, 370 healthcare workers were administered structured questionnaires. This was followed by in-depth interviews with a sub-sample of respondents to further clarify the responses regarding support for the coronavirus disease 2019 (COVID-19) vaccine mandate. Findings demonstrated that less than one-half of respondents supported the COVID-19 mandate, and only one in three had received the recommended COVID-19 vaccine doses. Support for the vaccine mandate and vaccine uptake were predicted by profession, work experience, number of children, health status, and risk perception. Support for the vaccine mandate was ascribed to ethical and professional duty, whereas opposition was associated with respect for autonomy and human rights. This study documents the need to enhance support for vaccine mandates and uptake among healthcare workers through sustainable strategies, as Nigeria’s healthcare workers are considered a source of trust and role models for the rest of society.

## 1. Introduction

Vaccine hesitancy, a leading global health threat, gained further notoriety following the discovery of COVID-19 vaccines [1,2]. Rumors and misinformation amplified by a vocal antivaccination movement discouraged the public and a sizeable proportion of health care professionals from taking the vaccine, threatening a return to normalcy [3,4,5]. Faced with the new SARS-CoV-2 variants and a surge in cases and deaths, many health authorities were forced to implement mandatory COVID-19 vaccination policies [6,7]. A vaccine mandate entails requiring vaccinations for working in settings such as health care and education or linking vaccine uptake to specific incentives or consequences, rather than forcibly vaccinating individuals [8].

Support for COVID-19 vaccine mandates among health care workers (HCWs) has ranged from 34% in Cyprus to 94% in Mongolia [9,10]. Similarly, among all workers, it ranged from 46% in Hungary to 97% in China, with a global average of 78% [11]. Furthermore, among members of the public, the figures varied from 40% across Africa to 73% in Australia [12,13]. COVID-19 vaccine uptake among HCWs also varies from 33% in Nigeria [14] to 77% in France [6] and 78% in China [15]. 

Predictors of support for vaccine mandates include sociodemographic (age, gender) and vaccine-related factors (knowledge, hesitancy, and uptake) [5]. Similarly, COVID-19 vaccine uptake is also influenced by knowledge of the disease and vaccines [16,17], risk perception, fear of COVID-19 [6], previous COVID test [17], vaccine-related concerns (efficacy, safety, side effects and fertility-related rumors) [16,17], motivation, and health service-related (ease of access to vaccination services) factors [14,18].

Understanding the attitude of HCWs towards COVID-19 vaccine mandates is important, considering their increased risk and likelihood of transmitting the virus to vulnerable populations. Health institutions have a legal and ethical duty to ensure a safe environment for staff and patients. By preventing severe COVID-19, vaccination sustains health system capacity through reduction in sickness absence, HCW hospitalizations, and ensuring the safety of vulnerable patients [19]. Furthermore, HCWs, as trusted sources of information, play a critical role in boosting public confidence in COVID-19 vaccines [20].

COVID-19 mandates have been implemented for federal HCWs in the US [21], Australia [22], Canada [23], and China [24]. Nigeria introduced a mandatory COVID-19 vaccination policy in December 2021, wherein all federal government employees were required to show evidence of COVID-19 vaccination or a negative COVID-19 PCR test result conducted within 72 h to gain access to their offices [25]. The acceptability of this policy among HCWs, who bridge the gap between health policymakers and the public, has not been well documented. Vaccination rates among HCWs correlate positively with their willingness to recommend COVID-19 vaccination to their patients [26]. Therefore, it is important to assess the support for this policy among HCWs, especially in northern Nigeria, where there is a demonstrated track record of resistance to vaccination programs, as highlighted during the polio eradication campaigns [27]. In this study we assess the predictors of support for COVID-19 vaccine mandate and vaccination status among HCWs in a tertiary hospital in Kano, Nigeria.

## 2. Methods

### 2.1. Study Area and Population

This study was conducted over a three-week period in February 2022 at the Aminu Kano Teaching Hospital (AKTH), a tertiary referral center serving more than 13 million people in Kano, Nigeria [28]. AKTH has a bed capacity of 750 and 3231 employees. Childhood immunization and COVID-19 vaccination services are available at the AKTH immunization clinic. 

The study population includes consenting AKTH staff providing clinical care and non-clinical staff providing administrative and support services. Eligible participants had to fulfill the following criteria: (1) employed at AKTH as clinical (physician, nurses/midwives, pharmacists, laboratory scientists, physiotherapists, community health officers, and ward attendants) or non-clinical staff (administrative, management, and support services), and (2) provided written informed consent. Staff on study/sick leave and those who withheld consent were excluded from the study. 

### 2.2. Study Design and Sampling

This was a sequential, explanatory, mixed-methods study deploying a pragmatic paradigm [29]. A structured survey was followed by in-depth semi-structured interviews with a sub-sample of respondents. The aim of the in-depth interviews was to illuminate the survey responses [30]. The target sample size for the survey was obtained using Fisher’s formula [31], assuming 50% support for COVID-19 vaccine mandates [32], 95% confidence level, and a 5% margin of error. The sample size (*n* = 384) was increased by 10% to account for non-response, giving a final sample size of 430. 

For the qualitative phase, a stratified purposive sub-sample of survey participants (*n* = 20) was interviewed to further clarify the responses regarding support for the COVID-19 vaccine mandate. We interviewed two survey respondents supportive of the COVID-19 vaccine mandate and two respondents opposed to the mandate from each of the clinical and non-clinical staff categories. 

### 2.3. Participant Recruitment and Sampling

We used a two-stage sampling method. In the first stage, the entire staff population (*n* = 3231) were categorized as physicians (*n* = 734), nurses/midwives (*n* = 798), other clinical (pharmacists, physiotherapists, laboratory scientists, community health officers) (*n* = 495), and non-clinical staff (administrative, management, support services) (*n* = 1204). Sample sizes were then allotted proportionate to stratum population with samples of 98, 107, 66, and 159 allocated to physicians, nurses/midwives, and other clinical and non-clinical staff, respectively. In stage two, after determining eligibility, systematic sampling was used to select participants in each category. After obtaining a sampling interval for each stratum, the first respondent was randomly selected between serial number 1 and the group’s sampling interval. Subsequent respondents were obtained by adding the group’s sampling interval to the previous respondent’s serial number. Sampled workers were then recruited into the study after providing detailed study information and obtaining informed consent.

### 2.4. Measures and Data Collection

For the survey, we adapted validated structured survey questionnaires from previous studies [5,17]. The first section documented socio-demographic characteristics including age, sex, marital status, ethnicity, education, religion, number of children, professional category, work experience, and general health status. The second section assessed self-perceived risk of COVID-19 using the question “How would you assess your chance of getting COVID-19?” The responses were (‘high’ or ‘low’), whether the respondent was worried about getting COVID-19, and whether the respondent had direct COVID-19 patient care responsibilities and documented previous COVID-19 tests. The third section determined COVID-19 vaccination status with the question “Have you been vaccinated against COVID-19 or not?” with “Yes” or “No” options. A follow-up question ascertained the type of vaccine and number of doses, including boosters for those that answered in the affirmative. Unvaccinated respondents were asked to list reasons for non-vaccination. The fourth section determined support for COVID-19 vaccine mandates for HCWs in Nigeria by asking “Do you oppose or support the (Nigerian) Federal Government policy of mandatory COVID-19 vaccination or regular PCR testing?” Supporters of the policy were asked their preference between mandatory vaccination and PCR testing. 

A pre-test was conducted on a 10% sample to assess the psychometric properties (re-validation and reliability) of the questionnaires at another hospital (Murtala Mohammed Specialist Hospital, Kano, Nigeria). All scales were reliable and sections consistent, with a Cronbach’s alpha of ≥0.80.

To clarify questionnaire responses, the in-depth interview guide had open-ended questions with probes for thick rich descriptions. The guide explored the motivations for supporting or opposing the COVID-19 vaccine mandate and the underpinnings of vaccine hesitancy among HCWs. All participants provided written informed consent. Confidentiality in reporting qualitative findings was maintained by removing identifiers. 

The study protocol was reviewed and approved by the Bayero University Research Ethics Committee. Potential participants were individually contacted by trained research assistants and provided detailed information on the study objectives and what participation entailed. They were informed that participation was voluntary and that they could skip any questions that they were not comfortable with. Those who signed an informed consent form were provided a self-administered questionnaire which was retrieved after completion. Two data entry clerks checked and independently entered the data in a password-protected database. To ensure confidentiality, serial numbers were assigned. Research assistants were trained in human research participant protection and the consent process.

#### 2.4.1. Statistical Analysis

Data were analyzed using SPSS Version 22 (IBM Corp., Armonk, NY, USA). Means and standard deviations were used to summarize numeric data. Frequencies and percentages were obtained for categorical variables. Pearson’s Chi-square or Fisher’s exact test was used as appropriate to assess the crude association between variables listed under the Measures and Data collection section and the two primary outcomes: (1) support for COVID-19 vaccine mandates, and (2) COVID-19 vaccination status [33]. Type I error was fixed at 5% for all tests.

Binary logistic regression models were developed for support for COVID-19 vaccine mandates and vaccination status. Independent variables with *p* < 0.10 at the bivariate level were included in the logistic regression models [34]. We selected the final model through a backward stepwise approach. Adjusted odds ratios (aORs) and their 95% confidence intervals (CIs) were obtained to measure the strength and direction of the effect of the independent variables on the outcome. The logistic model for predictors of health care workers’ support for COVID-19 vaccination-or-testing mandates includes the following variables: respondent’s sex, age group, ethnicity, professional category, years of experience, number of children, direct COVID-19 patient care, previous COVID-19 test, self-assessed health status, COVID-19 risk perception, concern about contracting COVID-19, vaccinated against COVID-19, considers COVID-19 vaccination an obligation, and awareness of vaccine recommendations for HCWs. Similarly, the logistic regression model for predictors of health care workers’ COVID-19 vaccination status include the variables: respondent’s sex, age group, marital status, professional category, years of experience, previous COVID-19 test, COVID-19 risk perception, concern about COVID-19 vaccine safety, concern about COVID-19 vaccine efficacy, concern about COVID-19 vaccine side effects, and concern about rumors related to infertility/depopulation. Hosmer-Lemeshow statistic and Omnibus tests were conducted to determine model fitness, with a Hosmer-Lemeshow chi-square yielding a *p*-value of >0.05, which is considered a good fit [35]. 

#### 2.4.2. Qualitative Data Analysis

Qualitative interviews were recorded and transcribed verbatim. A thematic analysis was performed based on the Framework Approach [36] and included familiarization through repeated reading, coding, theme generation, applying the codes to the transcripts, matrix formation, and interpretation. Findings from the two components of the mixed methods study were integrated [37].

## 3. Results

Of the 430 health workers approached, (86.0%, *n* = 370) completed the questionnaires. Less than half of the respondents (43.0%, *n* = 159) were female and the overall mean age (±standard deviation) was 35.7 ± 8.41. Physicians, nurses/midwives, and other clinical and non-clinical staff comprised 24.9%, 27.0%, 16.8%, and 31.4% of respondents, respectively. One-quarter (25.7%, *n* = 95) of the respondents had direct COVID-19 patient care responsibility. Of those ever tested for COVID-19 (*n* = 149), 14.9% (*n* = 55) tested positive (Table 1).

### 3.1. COVID-19 Risk Perception, Support for Vaccine Mandate, and Vaccination Status

Approximately one in five respondents (21.9%, *n* = 81) felt they were at high-risk for COVID-19 infection and one-half (52.2%, *n* = 193) were worried about contracting COVID-19, while more than three quarters (78.4%, *n* = 290) viewed COVID-19 complications as serious. Over half (55.7%, *n* = 206) of the respondents considered vaccination a collective responsibility and beneficial to all, while (44.3%, *n* = 164) regarded vaccination as an individual choice. About two-thirds (63.2%, *n* = 234) of the respondents had heard of the Nigerian government’s COVID-19 vaccination-or-testing mandate for federal employees, but less than half (47.8%, *n* = 177) of respondents supported the policy. Over one-half (52.2%, *n* = 193) of the respondents preferred periodic testing and masking over COVID-19 vaccination, and 5.4% (*n* = 20) would rather quit their jobs than get vaccinated. 

Overall, 35.9% (*n* = 133) of the respondents had received the recommended doses of COVID-19 vaccines. However, only 14.9% (*n* = 55) had received a booster dose. At the time of the study, 6.2% (*n* = 23) of respondents had received only one dose of a multi-dose COVID-19 vaccine, while over half (57.8%, *n* = 214) had not received any COVID-19 vaccines. The main reasons for vaccine hesitancy included concern about vaccine safety (13.5%, *n* = 50), fear of long-term effects (13.5%, *n* = 50), and low COVID-19 risk perception (10.5%, *n* = 39) (Table 2).

### 3.2. Predictors of Attitude towards COVID-19 Vaccine Mandate and Vaccine Uptake 

At the bivariate level, attitudes toward COVID-19 vaccine mandate and vaccination status were associated with respondents’ profession, work experience, number of children, and self-assessed health status (*p* < 0.05). Factors uniquely associated with attitude towards COVID-19 vaccine mandate included ethnicity, COVID-19 patient care, concerns about contracting COVID-19, vaccination status, and awareness of vaccinations recommended for HCWs. Additional factors associated with COVID-19 vaccination status included marital status, previous COVID-19 test, COVID-19 risk perception, and attitude towards vaccine mandates.

At the multivariate level, both attitudes towards COVID-19 vaccine mandates and vaccination status were independently predicted by the respondent’s profession, work experience, number of children, self-assessed health status, and COVID-19 risk perception. In addition, attitude toward the COVID-19 vaccine mandate was predicted by COVID-19 patient care, concerns about contracting COVID-19, and awareness of vaccines recommended for HCWs. Additional predictors for COVID-19 vaccination status included previous COVID-19 testing and attitude towards vaccine mandate.

Compared to physicians, nurse/midwives were 44% (adjusted odds ratio (aOR) = 0.56, 95% confidence interval (CI), 0.26–0.94) less likely to support COVID-19 vaccine mandates, while the non-clinical staff were twice as likely (aOR = 2.16, 95% CI, 1.15–4.44) to support the same. More experienced HCWs (5–9 years) were 40% (aOR = 0.60, 95% CI, 0.31–0.91) less likely to support COVID-19 vaccine mandates compared to less experienced colleagues (<5 years). Relative to nulliparous HCWs, their counterparts with at least one child were 76% (aOR = 0.24, 95% CI, 0.09–0.61) less likely to endorse COVID-19 vaccine mandates. Similarly, HCWs who did not provide COVID-19 clinical care were 51% (aOR = 0.49, 95% CI, 0.24–0.98) less likely to support vaccine mandates. Furthermore, HCWs who self-assessed their health status as good were 41% (aOR = 0.59, 95% CI, 0.35–0.96) less likely to support vaccine mandates. HCWs who perceived a low risk of acquiring COVID-19 were 48% (aOR = 0.52, 95% CI, 0.26–0.92) less likely to support vaccine mandates compared to those concerned about contracting COVID-19. Finally, HCWs who were fully vaccinated against COVID-19 (aOR = 2.06, 95% CI, 1.20–3.53) and those aware of vaccine recommendations for HCWs (aOR = 2.62, 95% CI, 1.59–4.33) had two-fold increased odds of supporting vaccine mandates (Table 3).

Regarding predictors of COVID-19 vaccination status, compared to physicians, nurse/midwives and other clinical/non-clinical staff were 36% (aOR =0.64, 95% CI, 0.30–0.92), 31% (aOR = 0.69, 95% CI, 0.31–0.88) and 46% (aOR = 0.54, 95% CI, 0.26–0.93) less likely to be vaccinated against COVID-19, respectively. Those that had 5–9 years’ work experience were 77% less likely to be vaccinated relative to those with <5 years’ work experience (aOR = 0.33, 95% CI, 0.16–0.69). Compared to nulliparous respondents, HCWs who had children were twice as likely (aOR = 2.19, 95% CI, 1.17–6.47) to have been vaccinated. Respondents previously tested for COVID-19 and those who perceived their risk as high had three-fold (aOR = 3.36, 95% CI, 1.91–5.91) and 50% (aOR = 1.50, 95% CI, 1.12–3.04) increased odds of being vaccinated, respectively. Finally, respondents with a favorable attitude towards vaccine mandates had two-fold (aOR = 2.07, 95% CI, 1.21–3.52) elevated odds of being vaccinated (Table 4).

### 3.3. Qualitative Findings

Themes from qualitative interviews include perception of a waning COVID-19 pandemic, complacency and response fatigue, vaccine hesitancy, and ambivalence toward the vaccine mandate policy.

#### 3.3.1. Risk Perception

Participants indicated that the COVID-19 pandemic was still a global threat with the occasional emergence of new variants. However, they were not as concerned as they were at the beginning of the pandemic. They indicated that COVID-19 cases were fewer and less severe as people became immune through vaccination or infection. 

“Though cases are fewer and less severe with better outcomes, COVID-19 remains a threat not only in Kano, or Nigeria, but globally due to the occasional emergence of new variants such as the delta and omicron variants. Who knows the strain recently ravaging the original source community in China could spread like wildfire again and we will be back in serious trouble.” Physician, 30-year-old.

#### 3.3.2. Complacency 

On whether HCWs still adhered to COVID-19 preventive measures, participants indicated that most HCWs were tired of the measures and were now complacent, but some had continued observing the precautions, although not necessarily because of COVID-19:

“…For me, because I have asthma, I still use the face mask not only for COVID-19 but as a dual purpose of also protecting against the harmattan dust that irritates the respiratory system leading to asthmatic attacks and other secondary respiratory infections.” Community health officer, 53-year-old.

#### 3.3.3. Vaccine Uptake

Some staff, especially doctors, were enthusiastic about the COVID-19 vaccination and most had taken the recommended doses, including the booster. However, most HCWs were still hesitant, and even those who had the vaccine never completed the recommended dosage due to safety concerns, rumors, and perceived invincibility:

“Vaccine uptake among hospital staff is still low. Some have received the recommended dose(s) of the COVID-19 vaccine including booster, while others are yet to do so. Some received a single dose and are afraid to complete the doses. I think they are still not convinced of the risk to the African and are concerned about vaccine safety from social media hype. They have a deep-seated belief that the vaccine is harmful and could cause problems in the long run.” Medical laboratory scientist, 52-year-old.

#### 3.3.4. Attitude towards Vaccine Mandates

There was a range of opinions regarding the Nigerian government’s COVID-19 vaccine mandates. Some participants voiced strong objections to the policy, citing ethical and human rights concerns:

“Most of the hospital workers are saying it is not right, because it is unethical to force someone to take the COVID-19 vaccine or any other vaccine against their wish. People have the right to decide about their health. Considering other medical problems that remain unaddressed, this is a misplaced priority by the government and is meant to please their international partners”. Medical laboratory scientist, 47-year-old.

Other participants supported vaccine mandates, stating that HCWs are societal role models, and so should be seen to practice what they preach. They opined that doing so will convince the public to accept the COVID-19 vaccine:

“…but for me, I support the policy of “operation show your COVID-19 vaccination card” among health workers. They should practice what they preach by accepting the COVID-19 vaccine. This is the only way that people will be convinced to take the jab.” Physician, 29-year-old.

Others anchored their support for the COVID-19 vaccine mandates to protecting the health workforce. They indicated that vaccinating frontline health workers is necessary to ensure health workers remained healthy enough to treat COVID-19 cases and other patients:

“It is a very good decision by the Federal Government. I am in support of it; it will reduce the hospital transmission and community spread of COVID-19. Health care workers, their families, and community members will be protected to provide health care for COVID patients and others.” Nursing officer, 47-year-old.

Some HCWs were unaware of the policy in Nigeria and in other countries. They suggested awareness creation as a strategy for improving vaccine uptake rather than enforcing vaccine mandates:

“I just heard of the mandatory requirement of COVID-19 vaccination by the Federal Government from you. I will say that they have undermined the autonomy of people to choose to be vaccinated, and there is nowhere in the world that the said vaccine is mandatory. I support awareness creation about the benefit of the vaccine instead of forcing people to take the vaccine.” Community health officer, 53-year-old.

#### 3.3.5. Reasons for Positions on Policy

Those who supported the vaccine mandate premised it on reducing the spread of the infection and ease of vaccination rather than periodic COVID-19 testing. If faced with the choice, they would prefer getting vaccinated to undergoing weekly COVID-19 testing, as they considered the latter cumbersome:

“Personally, I would rather take the vaccine than undergo weekly testing, because vaccines are now available in many places, but the PCR test is cumbersome and reagents will soon be out-of-stock.” Nursing officer, 47-year-old.

In contrast, participants’ opposition to the vaccine mandate was anchored to perceived misplaced priority, ethical and vaccine safety concerns, rumored side effects, unknown long-term effects, and human rights violations. 

“…Because there are other diseases of higher burden, morbidity and mortality than COVID-19, but the government is not talking about those. For example, where is the Malaria vaccine for our children?” Physiotherapist, 32-year-old.

Some HCWs would rather choose weekly PCR testing than vaccination because of the conspiracy theories and rumors relating the COVID-19 vaccine to infertility, electromagnetic effects at the vaccination site, and perceived economic motives underlying the speedy approval of COVID-19 vaccines. Others cited blood clots and deaths reportedly associated with vaccination in some high-income countries:

“…there are rumors that the vaccine was produced to deliberately depopulate Africa. Western countries rejected the initial vaccines and exported them to us, while providing themselves with the safer vaccines. Others believe that the vaccine is created by pharmaceutical companies to make money. There are also insinuations that the vaccination site becomes magnetic and is used by tech companies to track every citizen worldwide. People also mentioned the deadly blood clots.” Medical laboratory scientist, 52-year-old.

As to whether HCWs would contemplate quitting their jobs because of the vaccine mandate, no participant considered that as an option due to the difficulty of securing employment. They would rather test the mandate in the industrial court:

“Rather than quit their jobs if they are forced to take the COVID-19 vaccine, some hard-liners and unionists would test the mandate in the industrial court or report it to the human rights commission for arbitration.” Administrator, 40-year-old.

#### 3.3.6. Ethical Responsibility

Some participants viewed COVID-19 vaccination as an ethical obligation among HCWs to protect themselves, co-workers, family, and patients: 

“Yes, they (HCWs) have ethical and professional responsibility to get vaccinated for their protection, and those of co-workers, patients and members of their families. Their behavior will also influence community members’ COVID-19 vaccine uptake.” Administrator, 35-year-old.

Others were strongly opposed to vaccine mandates for HCWs based on ethical or professional obligations:

“Honestly, I don’t know if there is an ethical and professional responsibility for health care professionals to get vaccinated for the protection of others against COVID-19. Everyone should exercise his human rights.” Medical laboratory scientist, 52-year-old.

## 4. Discussion

This study assessed support for the COVID-19 vaccine mandate, vaccine uptake and predictors among HCWs in Nigeria. Over half of the respondents supported the policy and one-third had received COVID-19 vaccines. Vaccine hesitancy was related to safety concerns and low-risk perception. Vaccine mandate supporters felt that vaccination is an ethical and professional duty, viewing HCWs as health behavior role models and vaccination champions. Opposition was anchored to respect for autonomy and protection against human rights violations. Support for vaccine mandate and vaccine uptake were predicted by profession, work experience, number of children, health status, and risk perception. Additional predictors of support for vaccine mandate included COVID-19 patient care responsibility, concerns about contracting COVID-19, and awareness of recommended vaccines for HCWs. Other predictors of vaccine uptake were COVID-19 testing and attitude towards vaccine mandate.

Support for COVID-19 vaccine mandates documented in our study (47.8%) was lower than figures reported for HCWs in Asia (93.7%) [10], but higher than reports from France (35%) [38] and Cyprus (34%) [9]. Compared to our figure, vaccine mandate average support was also higher among all workers globally (78%), ranging from 46% in Hungary to 97% in China [11]. However, public support was lower across Africa (40%) [12] and the US (40.9%) [7], but higher in Europe (50–58%) [6,32] and Australia (73%) [13]. Differences in study populations, methods, and timing could partly account for these differences between our report and those of others. Other factors that could explain these disparities include variations in COVID-19 burden, risk perception, political ideology, trust in government, rumors, concerns about vaccine safety, philosophy, and human rights considerations. It is paradoxical that health care professionals, who should be well informed, are less supportive of the COVID-19 vaccine mandates compared to other workers and the public. This is a worrisome finding, as it could create doubt among other community members, thereby fueling vaccine hesitancy. Focusing on continuing professional education, trust, and confidence-building could improve the much-needed support for COVID-19 vaccination [39].

Regarding the possibility of quitting their jobs because of COVID-19 vaccine mandates, a lower proportion of our respondents (5.4%) would choose that path compared to the global figure (20%) [11]. This finding could be due to employment prospects, conviction against vaccine mandates, the legal milieu, and COVID-19 burden and risk perception.

The reasons underpinning HCWs’ support for vaccine mandates (ethical, professional obligation, societal health behavior models and vaccination champions) is consistent with findings from previous studies [19]. Likewise, vaccine mandate opposition stemming from ethical, legal, and human rights considerations have been reported [40,41]. Conspiracy theories, rumors and misinformation, mistrust for authorities and pharmaceutical companies, and political persuasion have all been documented [42,43]. In the UK, opposition to vaccine mandates has been based on respect for autonomy and workers’ moral integrity, the possible disproportionate effect of mandates on workers from minority ethnicities, which could in turn negatively affect service delivery, and the evolving definition of what constitutes ‘fully vaccinated’ for COVID-19 [44].

Our rate of COVID-19 vaccine uptake among HCWs (35.9%) is comparable to reports from other parts of Nigeria (33.3%) [14], but lower than findings by others (64.6%) [45]. Our uptake is also lower than rates in Asia (67.2–76.98%) [10,15], Europe (76.9%) [6], and the US (50–96%) [46]. Possible explanations for these variations include disparities in study population, timeframe, methods and measurement, vaccine knowledge, vaccine confidence, safety, risk perception and the enforcement of mandates. The reasons for low vaccine uptake in our sample (safety and fear of long-term unknown effects) are similar to findings in previous studies [16,17]. For instance, it was rumored that the COVID-19 vaccine contains surreptitious microchips that alter the recipient’s DNA [47]. These, combined with feelings of invincibility, the waning pandemic, and the relative sparing of parts of Africa have led to complacency. 

Variations in knowledge about vaccine development among the health care professions and in experiences with new vaccines could partly explain the predictive role of the respondents’ profession on vaccine uptake and support for mandates [17]. However, knowledge deficit alone is unlikely to explain the difference in vaccine uptake among health care professionals, as a study in Saudi Arabia in the early phase of the COVID-19 pandemic reported that the more educated were less likely to be vaccinated against COVID-19 [48]. HCWs of higher parity could have encountered new vaccines as part of the childhood immunization schedule and hence are more likely to support the COVID-19 vaccine mandate. Likewise, health status and COVID-19 risk perceptions could influence vaccine uptake and support, whereby individuals who feel invincible are likely to be vaccine-hesitant [6]. Furthermore, frontline HCWs exposed to the risk of contracting COVID-19 are more likely to get vaccinated and support mandates. Previous COVID-19 tests could be a proxy for exposure—persons who have been tested are therefore likely to receive the vaccine. Similarly, support for the vaccine mandate is likely among vaccinated HCWs who have been convinced about the safety and efficacy of the vaccines. 

Our findings have implications for practice. With the continuous threat of emergence and re-emergence of infectious disease pandemics, it is imperative to sustain strategies to enhance the support of immunization programs among HCWs. First, the sizeable minority of HCWs that were unaware of the government’s vaccine mandate policy two months into its implementation clearly indicates a communication gap between policymakers, health managers, and HCWs. There is a need to bridge this communication gap and the disparity between support for the vaccine mandate and COVID-19 vaccine uptake among HCWs. The focus should be on sustaining continuous professional development, access to cutting-edge scientific information, the inclusion of vaccinology courses in pre-qualification curricula of the training institutions, and critical tools to counter fake news, rumors, and conspiracy theories, especially in social media. In addition, measures to increase access to COVID-19 vaccination, elimination of obstacles and barriers to vaccination, the creation of messages to counter misinformation, and educational campaigns about vaccine safety and the societal benefits of high vaccination coverage should be emphasized. 

Our study is one of the first to investigate attitudes toward the COVID-19 vaccine mandate among HCWs in Nigeria. We deployed a mixed methods approach, with qualitative in-depth interviews providing a nuanced explanation of motives, thoughts, and reasons for support or opposition to the mandate. The study was also conducted when COVID-19 vaccines were available and soon after the effective date of the policy. Nonetheless, the study had limitations that could be addressed in subsequent studies. First, the cross-sectional design precludes the attribution of causality between observed phenomena. Second, the conduct of the study at one tertiary health institution calls for caution when generalizing findings. Third, interview subjects readily offered group viewpoints rather than individual perspectives about risk perception, complacency and support for vaccine mandates. This could be a social desirability bias related to the local culture or worry about authorities taking action. Given the influence of professional role, years of experience, parity, and self-assessed health from the survey, we request a more detailed qualitative study focusing on the opinions of study participants rather than group views in these categories, as it could provide insight into possible cultural or class dimensions to the difference between attitude and uptake of COVID-19 vaccines between physicians, nurses/midwives, and other HCWs. Future studies should also track the attitude of HCWs towards mandatory vaccination, taking into consideration their influence on patients and the public.

## 5. Conclusions

We set out to determine the predictors of support for COVID-19 mandates and vaccine uptake among HCWs. Support for COVID-19 vaccine mandates was moderate and COVID-19 vaccine uptake sub-optimal. They were influenced by professional experience, risk perception, parity, self-assessed health status, COVID-19 patient care, previous testing, and knowledge of recommended vaccines for HCWs. Multiple strategies including continuous professional education, trust building, search for cultural influences and evidence-based counter-messaging are required to increase confidence, boost support, and improve the uptake of COVID-19 vaccines in similar settings. 

## Figures and Tables

**Table 1:** Characteristics of health care workers, Kano, Nigeria, 2022.

Characteristics	Frequency No. (%) *N* = 370
*Sex*	
Male	211 (57.0)
Female	159 (43.0)
*Age group*	
<30	104 (28.1)
30–39	155 (41.9)
≥40	111 (30.0)
*Ethnicity*	
Hausa/Fulani	287 (77.6)
Others *	83 (22.4)
*Religion*	
Islam	328 (88.7)
Christianity	42 (11.4)
*Marital status*	
Single	80 (21.6)
Ever Married	290 (78.4)
*Professional category*	
Physician	92 (24.9)
Nurse/Midwife	100 (27.0)
Other Clinical **	62 (16.8)
Non-Clinical	116 (31.4)
*Years of experience*	
<5	147 (39.7)
5–9	88 (23.8)
≥10	135 (36.5)
*No. of children*	
0	100 (27.0)
1	40 (10.8)
2–4	158 (42.7)
≥5	72 (19.5)
*Has chronic medical disorder*	
Yes	50 (13.5)
No	320 (86.5)
*Self-assessed health status*	
Very good	206 (55.7)
Good	148 (40.0)
Fair	16 (4.3)
*Has direct COVID-19 patient care responsibilities*	
Yes	95 (25.7)
No	275 (74.3)

* Other ethnicities include Yoruba, Igbo, Kanuri, Egbira, Igala. ** Other clinical staff include pharmacists, physiotherapists, laboratory scientists, community health officers.

**Table 2:** COVID 19-risk perception, vaccination, and testing (*N* = 370).

	Frequency *n* (%)
***COVID-19 Risk perception***	
Feels his/her chance of getting COVID-19 is high	81 (21.9)
Worried about getting COVID-19	193 (52.2)
Perceives complications of COVID-19 as serious	290 (78.4)
***Previous COVID-19 testing***	
Ever tested for COVID-19	149 (40.3)
Tested positive for COVID-19	55 (14.9)
***COVID-19 vaccination status***	
Have received the recommended dose(s) of COVID-19 vaccine plus booster	55 (14.9)
Have received the recommended dose(s) of COVID-19 vaccine but not the booster dose	78 (21.1)
Have received only one dose of a multi-dose COVID-19 vaccine	23 (6.2)
Have not received the COVID-19 vaccine at all	214 (57.8)
***Reasons for non-vaccination (multiple responses)***	
Concerned about safety as the vaccine was developed too quickly	50 (13.5)
Fear of long-term effects of the vaccine	50 (13.5)
Low COVID-19 risk perception hence doesn’t need the vaccine	39 (10.5)
Against vaccination in general	30 (8.1)
Medical reason for not taking the vaccine	17 (4.6)
Concerned about infertility-related rumors	15 (4.1)
A religious reason for not taking the vaccine	11 (3.0)
Others	19 (5.1)
***Perceptions about vaccination in general***	
Vaccination is an individual’s choice	164 (44.3)
Vaccination is a collective responsibility for the health of all	206 (55.7)
HCWs should get vaccinated to protect themselves, family and patients	307 (83.0)
***Support for COVID-19 vaccine-or-/testing mandates for HCWs***	
Aware of Nigeria’s national government COVID-19 vaccination-or-testing mandate	234 (63.2)
Supports the Nigerian government’s COVID-19 vaccination-or-testing mandates	177 (47.8)

**Table 3:** Logistic regression model for predictors of health care workers’ support for COVID-19 vaccination-or-testing mandate, Kano, Nigeria (*N* = 370).

Characteristics	*N*	Proportion of Health Workers’ Who Support the COVID-19 Vaccine Mandate No. (%)	*p*-Value	Crude OR (95% CI)	Adjusted OR (95% CI)	*p*-Value
***Sex***			0.52			
Male	211	104 (49.3)		1.15 (0.76–1.73)	1.07 (0.62–1.84)	0.71
Female	159	73 (45.9)		Referent	Referent	
***Age group***			0.063			
<30	104	55 (50.9)		Referent	Referent	
30–39	155	63 (40.7)		0.61 (0.37–1.01)	0.56 (0.26–1.18)	0.19
≥40	111	59 (53.2)		1.01 (0.59–1.73)	0.63 (0.23–1.73)	0.96
***Ethnicity***			0.03 *			
Hausa/Fulani	287	146 (50.9)		1.74 (1.15–2.87)	1.02 (0.55–1.89)	0.63
Others *	83	31 (37.4)		Referent	Referent	
***Religion***			0.18			
Islam	328	161 (49.1)		--	--	
Christianity	42	16 (38.1)		--	--	
***Marital status***			0.49			
Single	80	41 (51.3)		--	--	
Ever Married	290	136 (46.9)		--	--	
***Professional Category***			0.003 *			
Physician	92	48 (52.2)		Referent	Referent	
Nurse/Midwife	100	32 (32.0)		**0.43 (0.24–0.78)**	**0.56 (0.26–0.94)**	0.024 *
Other Clinical **	62	32 (51.6)		0.98 (0.51–1.86)	1.17 (0.52–2.63)	0.27
Non-clinical staff	116	65 (56.0)		**1.17 (1.05–2.02)**	**2.16 (1.15–4.44)**	0.011 *
***Years of experience***			0.016 *			
<5	147	80 (54.4)		Referent	Referent	
5–9	88	31 (35.2)		**0.46 (0.26–0.79)**	**0.60 (0.31–0.91)**	0.032 *
≥10	135	66 (48.9)		0.80 (0.50–1.28)	0.78 (0.36–1.68)	0.51
***Number of children***			0.039 *			
0	100	53 (53.0)		Referent	--	
1	40	11 (27.5)		**0.34 (0.15–0.75)**	**0.24 (0.09–0.61)**	0.009 *
2–4	158	76 (48.1)		0.82 (0.50–1.36)	0.89 (0.45–1.80)	0.86
≥5	72	37 (51.4)		0.94 (0.51–1.72)	0.91 (0.35–2.34)	0.81
***Direct COVID-19 patient care responsibilities***			0.012 *			
Yes	95	56 (59.0)		Referent	Referent	
No	275	121 (44.0)		**0.55 (0.34–0.88)**	**0.49 (0.24–0.98)**	0.027 *
***Ever tested for COVID-19***			0.43			
Yes	149	75 (50.3)		Referent	Referent	
No	221	102 (46.2)		0.85 (0.56–1.28)	0.89 (0.49–1.59)	0.52
***History of chronic medical disorder***			0.74			
Yes	50	25 (50.0)		--	--	
No	320	152 (47.5)		--	--	
***Self-assessed health status***			0.003 *			
Very Good	206	105 (51.0)		Referent	Referent	
Good	148	59 (39.9)		**0.64 (0.42–0.98)**	**0.59 (0.35–0.96)**	0.026 *
Fair	16	13 (81.3)		**4.17 (1.15–15.06)**	**3.41 (1.17–15.9)**	0.029 *
***COVID-19 risk perception***			0.068			
High	81	46 (56.8)		Referent	Referent	
Low	289	131 (45.3)		**0.63 (0.38–0.89)**	**0.52 (0.26–0.92)**	0.031 *
***Concerned about contracting COVID-19***			0.001 *			
Yes	193	109 (56.5)		**2.08 (1.37–3.15)**	**1.56 (1.24–3.52)**	0.038 *
No	177	68 (38.4)		Referent	Referent	
***Vaccinated against COVID-19***			<0.001 *			
Yes	152	90 (51.2)		**2.18 (1.43–3.33)**	**2.06 (1.20–3.53)**	0.019 *
No	218	87 (39.9)		Referent	Referent	
***Considers HCW vaccination an obligation to protect self, family, and patients***			<0.001 *			
Yes	307	169 (55.1)		**8.42 (3.88–18.27)**	**6.37 (2.66–15.23)**	<0.001 *
No	63	8 (12.7)		Referent	Referent	
***Aware of vaccine recommendations for HCWs***			<0.001 *			
Yes	191	114 (59.7)		**2.73 (1.79–4.16)**	**2.62 (1.59–4.33)**	0.003 *
No	179	63 (35.2)		Referent	Referent	

* Significant at *p* < 0.05; OR: Odds Ratio, CI: confidence interval; Hosmer-Lemeshow Chi-square = 7.82, *p* = 0.45. The logistic model includes the following variables: Respondent’s sex, age group, ethnicity, professional category, years of experience, number of children, direct COVID-19 patient care, previous COVID-19 test, self-assessed health status, COVID-19 risk perception, concern about contracting COVID-19, vaccinated against COVID-19, considers COVID-19 vaccination an obligation, and awareness of vaccine recommendations for HCWs. ** Other clinical staff include pharmacists, physiotherapists, laboratory scientists, com-munity health officers.

**Table 4:** Logistic regression model for predictors of health care workers’ COVID-19 vaccination status, Kano, Nigeria (*N* = 370).

Characteristics	*N*	The Proportion of Health Care Workers Vaccinated † Against COVID-19 No. (%)	*p*-Value	Crude OR (95% CI)	Adjusted OR (95% CI)	*p*-Value
***Sex***			0.36			
Male	211	80 (37.9)		1.22 (0.79–1.88)	1.13 (0.65–1.96)	0.87
Female	159	53 (33.3)		Referent	Referent	
***Age group***			0.18			
<30	104	30 (28.9)		Referent	Referent	
30–39	155	58 (37.4)		1.47 (0.86–2.52)	1.34 (0.61–2.93)	0.31
≥40	111	45 (40.5)		1.68 (0.95–2.97)	2.23 (0.79–6.28)	0.38
***Ethnicity***			0.46			
Hausa/Fulani	287	106 (36.9)		--	--	
Others *	83	27 (32.5)		--	--	
***Religion***			0.08			
Islam	328	123 (37.5)		1.92 (0.91–4.04)	1.55 (0.61–3.91)	0.63
Christianity	42	10 (23.8)		Referent	Referent	
***Marital status***			0.01 *			
Single	80	19 (23.8)		Referent	Referent	
Ever Married	290	114 (39.3)		2.08 (1.18–3.66)	1.08 (0.42–2.81)	0.89
***Professional Category***			0.014 *			
Physician	92	46 (50.0)		Referent	Referent	
Nurse/Midwife	100	32 (32.0)		**0.47 (0.26–0.82)**	**0.64 (0.30–0.92)**	0.027 *
Other Clinical **	62	20 (32.3)		**0.48 (0.24–0.93)**	**0.69 (0.31–0.88)**	0.034 *
Non-clinical staff	116	35 (30.2)		**0.43 (0.24–0.76)**	**0.54 (0.26–0.93)**	0.018 *
***Years of experience***			0.007 *			
<5	147	52 (35.4)		Referent	Referent	
5–9	88	21 (23.9)		**0.57 (0.32–0.97)**	**0.33 (0.16–0.69)**	0.021 *
≥10	135	60 (44.4)		1.46 (1.10–2.36)	1.20 (0.54–2.70)	0.56
***Number of children***			0.001 *			
0	100	27 (27.0)		Referent	Referent	
1	40	18 (45.0)		**2.21 (1.13–4.75)**	**2.19 (1.17–6.47)**	0.016 *
2–4	158	72 (45.6)		**2.26 (1.32–3.89)**	**2.03 (1.14–5.19)**	0.025 *
≥5	72	16 (22.2)		0.77 (0.38–1.57)	0.32 (0.10–1.02)	0.64
***Direct COVID-19 patient care responsibilities***			0.48			
Yes	95	37 (38.9)		--	--	
No	275	96 (34.9)		--	--	
***Ever tested for COVID-19***			<0.001 *			
Yes	149	74 (49.7)		**2.71 (1.75–4.20)**	**3.36 (1.91–5.91)**	<0.001 *
No	221	59 (26.7)		Referent	Referent	
***Self-assessed health status***			0.011 *			
Very Good	206	66 (32.0)		Referent	Referent	
Good	148	56 (37.8)		**1.29 (0.83–2.01)**	**1.43 (1.10–2.39)**	0.035 *
Fair	16	11 (68.8)		**4.67 (1.56–13.98)**	**4.50 (1.23–16.52)**	0.017 *
***COVID-19 risk perception***			0.01 *			
High	81	39 (48.2)		**1.93 (1.17–3.18)**	**1.50 (1.12–3.04)**	0.034 *
Low	289	94 (32.5)		Referent	Referent	
***Considers HCW vaccination an obligation to protect self, family, and patients***			<0.001 *			
Yes	307	128 (41.7)		**8.30 (3.24–21.26)**	**6.19 (2.22–17.24)**	0.004 *
No	63	5 (7.9)		Referent	Referent	
***Attitude towards a national policy on COVID-19 vaccine mandates***			0.002 *			
Positive	177	78 (44.1)		**1.98 (1.28–3.04)**	**2.07 (1.21–3.52)**	0.019 *
Negative	193	55 (28.5)		Referent	Referent	

† Hospital workers that reported receiving recommended doses with or without booster doses, * Significant at *p* < 0.05; OR: odds ratio, CI: confidence interval, Hosmer-Lemeshow Chi-square = 10.7, *p* = 0.22, The logistic model includes the following variables: respondent’s sex, age group, marital status, professional category, years of experience, previous COVID-19 test, COVID-19 risk perception, concern about COVID-19 vaccine safety, concern about COVID-19 vaccine efficacy, concern about COVID-19 vaccine side effects, and concern about rumors related to infertility/depopulation. ** Other clinical staff include pharmacists, physiotherapists, laboratory scientists, com-munity health officers.

## Data Availability

Data will be made available on a case-by-case basis and in accordance with Nigeria data privacy laws.
